# Data for engineering lipid metabolism of Chinese hamster ovary (CHO) cells for enhanced recombinant protein production

**DOI:** 10.1016/j.dib.2020.105217

**Published:** 2020-01-31

**Authors:** James D. Budge, Tanya J. Knight, Jane Povey, Joanne Roobol, Ian R. Brown, Gurdeep Singh, Andrew Dean, Sarah Turner, Colin M. Jaques, Robert J. Young, Andrew J. Racher, C Mark Smales

**Affiliations:** a Industrial Biotechnology Centre, School of Biosciences, University of Kent, Canterbury, Kent CT2 7NJ, UK; b Lonza Biologics, 228 Bath Road, Slough, SL1 4DX, UK; c Cell Engineering Group, Lonza Biologics, Granta Park, Cambridge, CB21 6GS, UK

**Keywords:** Chinese hamster ovary (CHO) cells, Lipid metabolism engineering, Cell line engineering, Difficult to express protein, Recombinant protein, Endoplasmic reticulum, Transcription factor engineering

## Abstract

The data presented in this article relates to the manuscript entitled ‘Engineering of Chinese hamster ovary cell lipid metabolism results in an expanded ER and enhanced recombinant biotherapeutic protein production’, published in the Journal Metabolic Engineering [1]. In the article here, we present data examining the overexpression of the lipid metabolism modifying genes *SCD1* and *SREBF1* in CHO cells by densitometry of western blots and by using mass spectrometry to investigate the impact on specific lipid species. We also present immunofluorescence data at the protein level upon SCD1 and SREBF1 overexpression. The growth profile data during batch culture of control CHO cells and CHO cells engineered to overexpress SCD1 and SREBF1 during batch culture are also reported. Finally, we report data on the yields of model secretory recombinant proteins produced from control, SCD1 or SREBF1 engineered cells using a transient expression systems.

**Table:** Specifications Table

Subject	Biotechnology
Specific subject area	Lipid Metabolism Metabolic Engineering and Recombinant Protein Expression from CHO Cells
Type of data	Table Image Chart Graph Figure
How data were acquired	Viable cell concentration and culture viability measurements were collected on a ViCell instrument (Beckman Coulter). Secreted recombinant protein concentrations in cell culture supernatants were determined using an Octet® instrument (ForteBio) with IgG calibrators and protein A biosensors. Western blot analysis was undertaken on standard laboratory equipment. Microscopy images were collected on a Zeiss LSM 880/Elyra/Axio Observer Z1 confocal microscope. Mass spectrometry data was collected on a Synapt G2Si (Waters) mass spectrometer. The data was analyzed using the Waters software UNIFI searching a Waters Lipid Maps database.
Data format	Raw; this manuscript, reference [3] and http://doi.org/10.5281/zenodo.3610075. doi: 10.5281/zenodo.3610075 Analyzed
Parameters for data collection	Control, *SCD1* and *SREBF1* engineered Chinese hamster ovary cells were cultured under standard batch conditions and transfected to transiently express a range of model secreted biotherapeutic recombinant proteins.
Description of data collection	Western blot and densitometry analysis was undertaken to investigate SCD1 and SREBF1 expression. Immunofluorescence analysis was undertaken to analyze expression and cellular localization of SCD1 and SREBF1. Cell counting was undertaking to determine viable cell numbers and culture viability during batch culture. An Octet® instrument and western blotting was used to estimate secreted recombinant protein yields. Mass spectrometry was undertaken to investigate specific lipids in control, SCD1 and SREBF1 engineered cells.
Data source location	Institution: University of Kent City/Town/Region: Canterbury Country: UK
Data accessibility	With the article, and/or in Refs. [1,3], and in the Zendo data repository; https://doi.org/10.5281/zenodo.3610075.
Related research article	Budge JD, Knight TJ, Povey J, Roobol J, Brown IR, Singh G, Dean A, Turner S, Jaques CM, Young RJ, Racher AJ, Smales CM (2020) Engineering of Chinese hamster ovary cell lipid metabolism results in an expanded ER and enhanced recombinant biotherapeutic protein production, *Metabolic Engineering*, 57:203–216. https://doi.org/10.1016/j.ymben.2019.11.007.

**Table:** 

**Value of the Data** • The data in this work is on the expression and cellular localization of exogenous SCD1 and SREBF1 in Chinese hamster ovary (CHO) cells, and the impact on the transient yield of model secretory biotherapeutic proteins from such engineered cells • The data reports the impact on the growth of SCD1 and SREBF1 engineered CHO cells during batch culture • Collectively, these data will benefit those interested in (a) expressing biotherapeutic recombinant proteins in high yields and quality from mammalian cells, and (b) understanding the role of manipulating lipid metabolism on the lipid composition of cells and the resulting cellular phenotypes • These data provide the basis for designing further and novel approaches based around modifying lipid metabolism in mammalian cells to improve such cells ability to produce higher yields of difficult to express biotherapeutic proteins

## 1 Data description

**Fig. 1:**
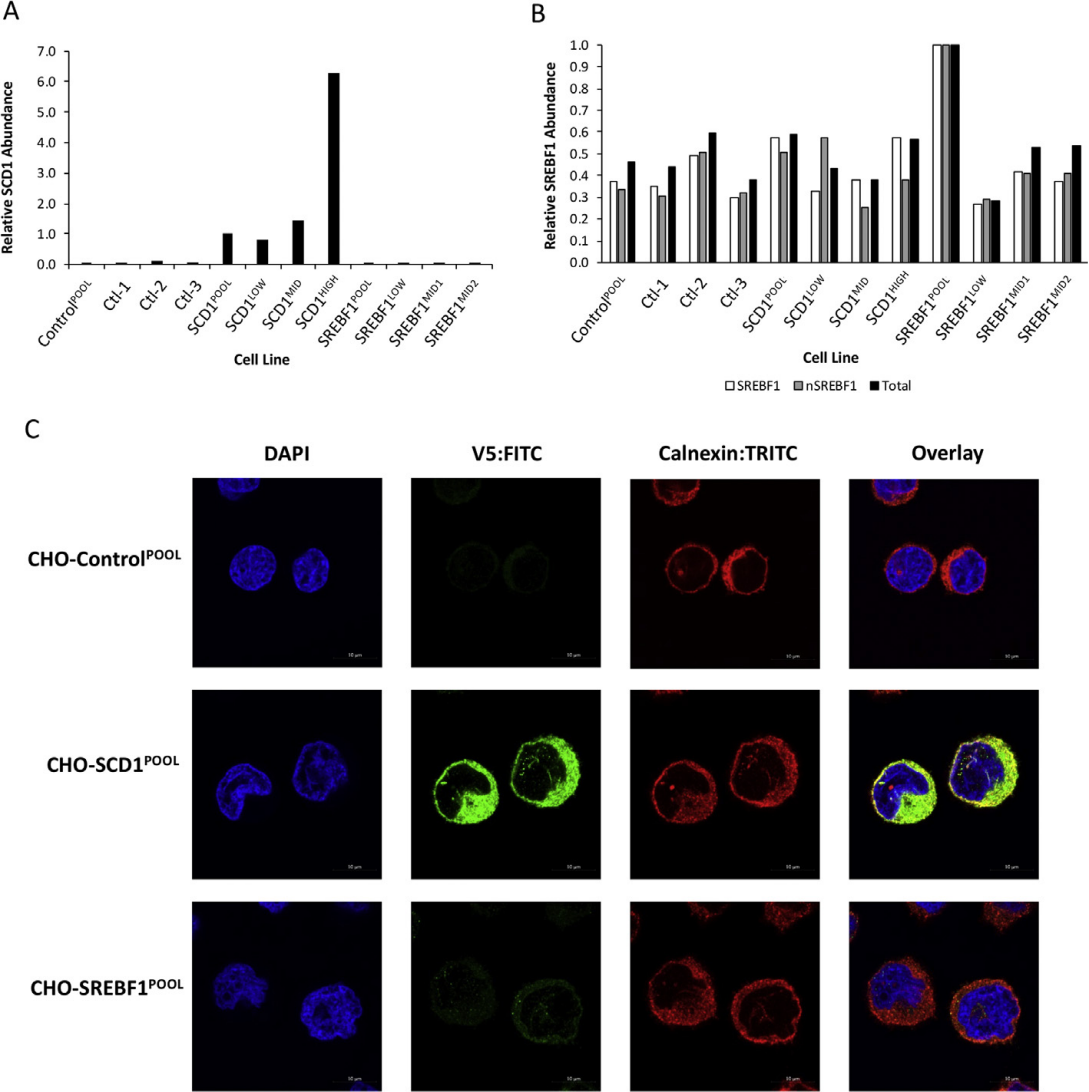
**Quantitative analysis of relative overexpression levels and cellular localization of SCD1 and SREBF1 in engineered cells**. Figure A shows the relative abundance of SCD1 as calculated using densitometry from the western blots reported in Fig. 2A and B of [1], whilst figure B shows the relative abundance of SREBF1 species. The values have been normalized to L7α loading control and subsequently normalized to either CHO-SCD1^POOL^ (A) or CHO-SREBF1^POOL^ (B) values. Cellular localization of overexpressed SCD1 and SREBF1 proteins in CHO-Control^POOL^, CHO-SCD1^POOL^ and CHO-SREBF1^POOL^ cell pools as determined by immunofluorescent detection using an anti-V5 antibody conjugated with a FITC secondary antibody (C). An anti-calnexin antibody conjugated with a TRITC secondary antibody was used to highlight the position of the ER. Images were obtained using confocal microscopy.

**Fig. 2:**
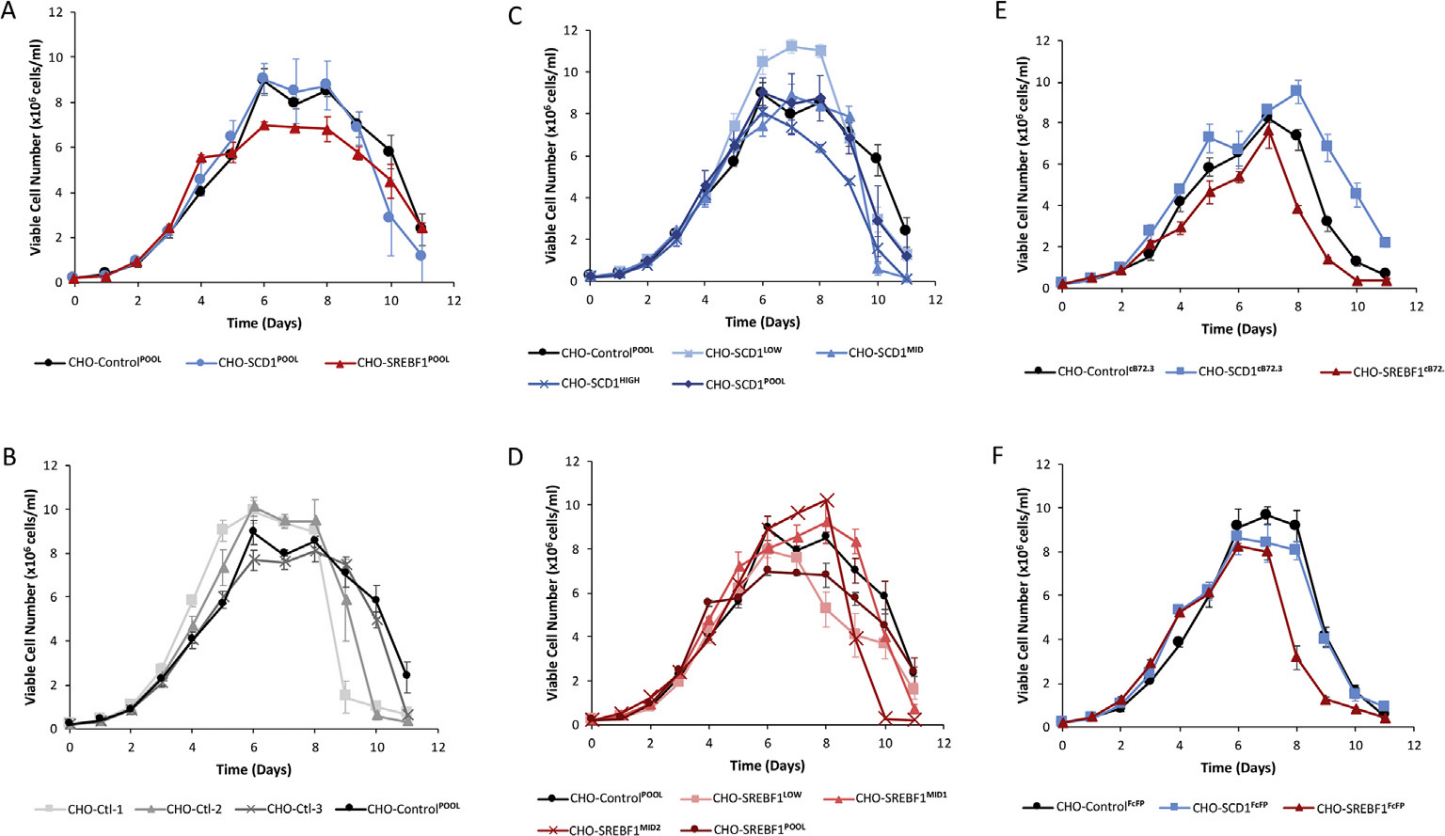
**Growth profiles of CHOK1SV GS-KO™ cells engineered to overexpress lipid metabolism modifying genes *SCD1* and *SREBF1***. Batch cultures were seeded at 0.2 × 10^6^ viable cells/ml in a total culture volume of 20 ml and cell concentrations measured using a ViCell every 24 h. The CHO-Control^POOL^ growth profile is shown in A-D for reference whilst lipid metabolism modified pool data are shown in (A), control monoclonal measurements are shown in (B), SCD1 monoclonal samples are shown in (C) and SREBF1 monoclonal measurements are shown in (D). Cell pools were constructed using lipid modified polyclonal cell pools as hosts such that they stably express either the cB72.3 monoclonal antibody or FcFP molecule. Growth profiles of these cells are shown in Figure (E) for cB72.3 expressing pools and Figure (F) for FcFP expressing pools. n = 3 for each data point and error bars show ± one standard deviation.

**Fig. 3:**
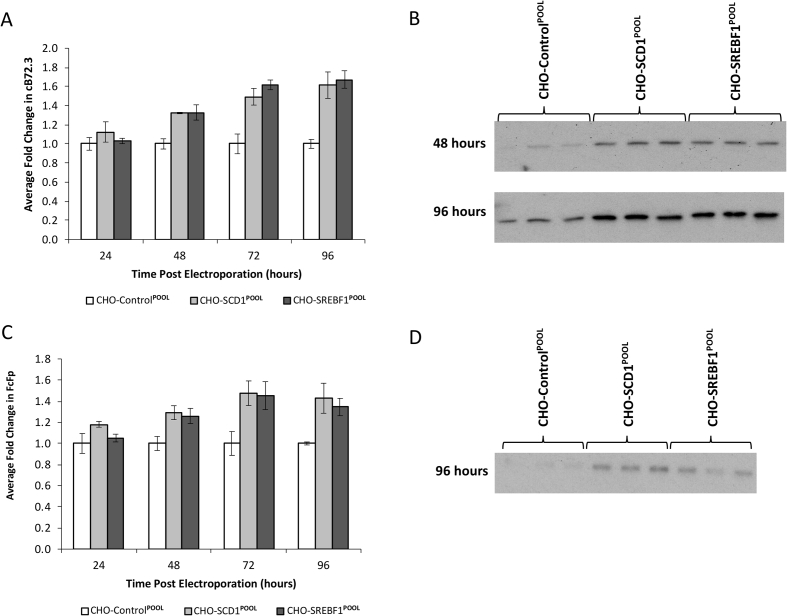
**Product analysis post transient transfection in lipid modified cells**. Previously engineered host cells (CHO-Control^POOL^, CHO-SCD1^POOL^, CHO-SREBF1^POOL^) were transiently transfected with vectors containing genes for expression of either cB72.3 (A and B) or FcFP (C and D). Quantitative analysis using an Octet® was carried out on supernatant samples harvested at 24, 48, 72 and 96 h post transfection for cB72.3 (A) and FcFP (C) transfections. Western blot analysis of cB72.3 (B) and FcFP (D) molecules were carried out on supernatant samples harvested at 96 h, and 48 h in the case of cB72.3. n = 3 for each data point and error bars show ± one standard deviation.

**Fig. 4:**
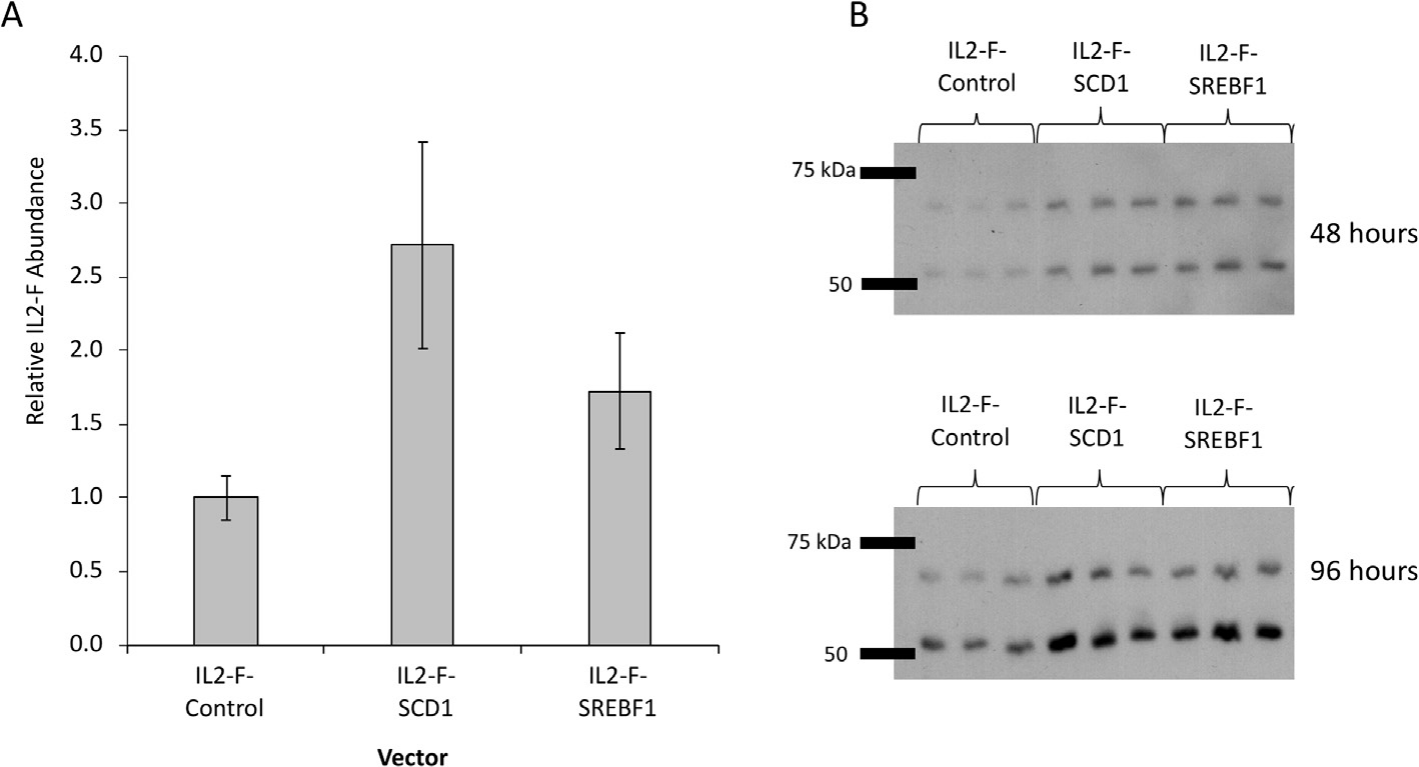
**Product analysis post transient transfection with vectors bearing genes for a recombinant product and a lipid modifying gene on the same expression vector**. Vectors containing genes necessary for expression of an antibody fusion molecule (IL2-F) with either no additional LMM gene (IL2-F-Control), the *SCD1* gene (IL2-F-SCD1) or the *SREBF1* gene (IL2-F-SREBF1) on the same vector. Quantitative analysis using an Octet® was carried out on supernatant samples harvested at 96 h post transfection to measure relative product concentration (A). Western blot analysis was also carried out on reduced samples. A heavy chain band (approximately 50 kDa) and an antibody heavy chain-IL2 fusion band (approximately 70 kDa) associated with the antibody fusion molecule is observed (B). n = 3 for each data point and error bars show ± one standard deviation.

**Fig. 5:**
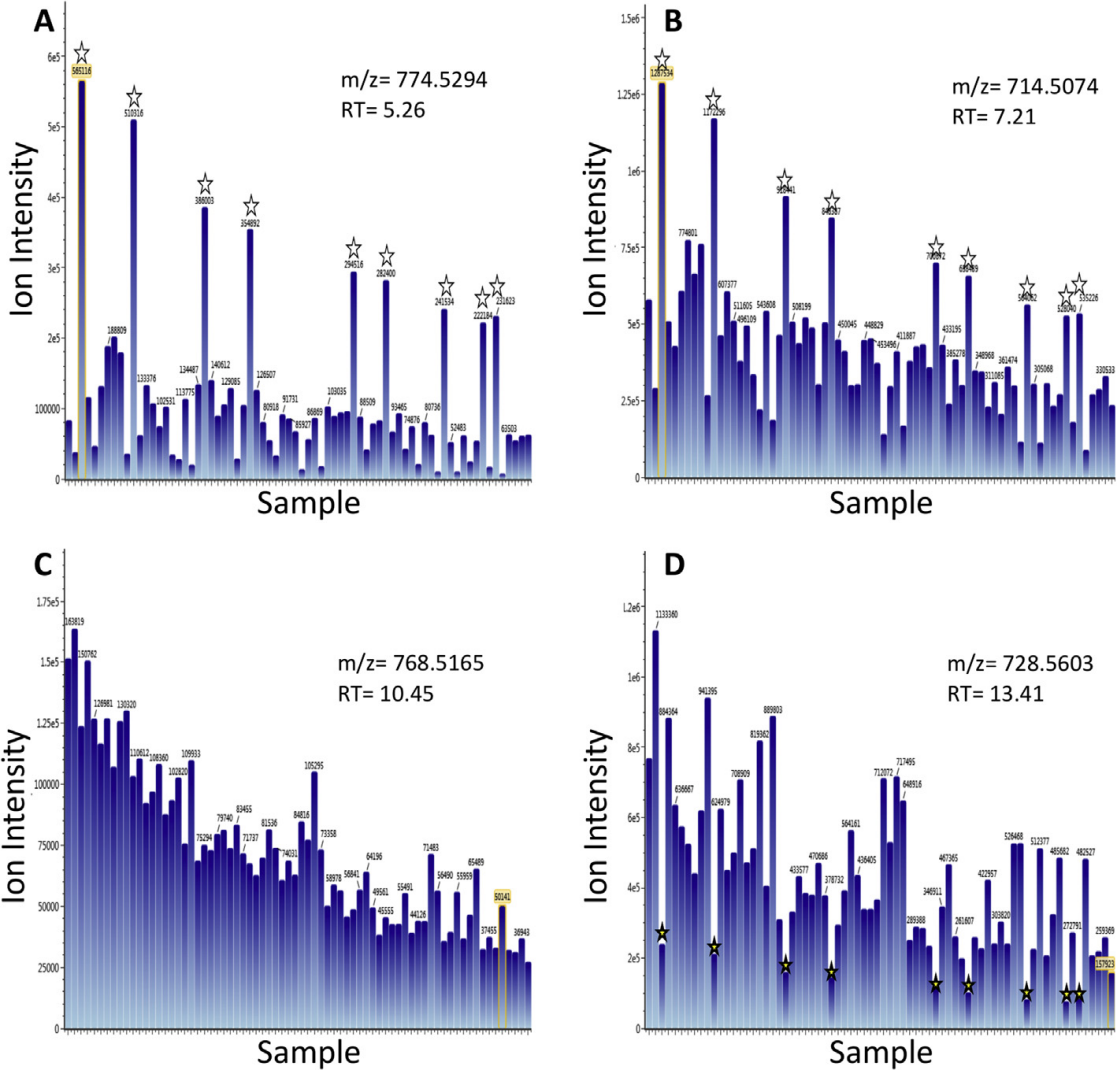
**Analysis of specific cellular lipids in control and LMM engineered CHO cells using mass spectrometry**. Figures A to D show relative quantities of specific lipid species as indicated by ion intensities at specific retention times. Stars represent data obtained from SCD1 high samples which are either upregulated (A and B), downregulated (D), or unchanged (C) compared to the control and other SCD1 engineered cell pools and clones. Retention times (RT) and *m*/*z* ions are indicated within the figure.

This article reports experimental data on the overexpression of the lipid metabolism modifying genes *stearoyl CoA desaturase 1* (*SCD1*) and *sterol regulatory element binding factor 1* (*SREBF1*) in Chinese hamster ovary (CHO) cells and the yields of model secretory recombinant proteins achieved from these during batch culture in a transient expression mode. A summary of vectors bearing genes for production of model recombinant biotherapeutic molecules used during this study is reported (Table 1). Details of antibodies used for analysis, including the manufacturer and reference, the organism produced in, its applications in the studies reported in this article and in Ref. [1], and the dilution used, are outlined in Table 2. Analysis of exogenous SCD1 and SREBF1 protein expression by densitometry of western blots, and the cellular localization by immunofluorescence, is reported in Fig. 1. The raw Western blot and microscopy image files can be found in the Zendo data repository [3]. The batch-culture growth profiles of control and SCD1 or SREBF1 engineered cells are shown along with the profiles when such cells are used to create stable model secretory recombinant protein expressing cell pools in Fig. 2. Analysis of transient secreted recombinant protein yields achieved from control and SCD1 and SREBF1 engineered CHO cell pools is analyzed in Fig. 3. Analysis of the yield of a protein considered by the authors to be difficult to express when transiently expressed in CHO cells alongside lipid metabolism modifying genes is shown in Fig. 4. Analysis of specific cellular lipids in control and lipid metabolism modifying engineered CHO cells using mass spectrometry is also reported in Fig. 5. The raw mass spectrometry data files can be found in the Zendo data repository ([3] doi: 10.5281/zenodo.3610075).

**Table 1:** A summary of vectors bearing genes for production of model recombinant biotherapeutic molecules used and in Budge et al. [1]. The type of recombinant molecule produced by the vector and number of genes required for expression of these molecules are outlined. The figures in which the vectors have been used and a brief description of the method by which they have been utilized are summarized.

Vector	Recombinant Molecule Type	No. of Genes	Brief Method
cB72.3	IgG4	2	Vector used to generate cells stably expressing cB72.3 molecule using hosts previously engineered to overexpress LMM molecules. Vector used to transiently produce cB72.3 molecule.
Fc-Fusion Protein	Fc fusion protein (FcFP)	1	Vector used to generate cells stably expressing an Fc fusion protein using hosts previously engineered to overexpress LMM molecules. Vector used to transiently produce an Fc fusion protein.
IL2-F-Control	Interleukin 2 fused bispecific antibody	3 plus LMM gene	Vectors bearing both IL2-F genes and LMM genes used to transiently co-transfect.
IL2-F-SCD1			
IL2-F-SREBF1			
IL-2-F	Interleukin 2 fused bispecific antibody	3	Vector used to generate cells stably expressing IL2-F molecule using hosts previously engineered to overexpress LMM molecules.
DTE-IgG1	IgG1	2	Vector used to generate cells stably expressing DTE-IgG1 molecule using hosts previously engineered to overexpress LMM molecules.

**Table 2:** Details of antibodies used for analysis including the manufacturer and reference, the organism produced in and its applications reported in this article and Budge et al. [1], and the dilution used.

Antibody	Company/Catalogue Number	Produced In	Use and Dilution
Primary Antibodies			
Anti-V5	Sigma/V8012	Mouse	Immunofluorescence- 1:500
Anti-calnexin	Abcam/ab22595	Rabbit	Immunofluorescence- 1:200
Anti-γ chain	Sigma/I9764	Rabbit	Western Blot- 1:2000
Anti-SCD1	Cell Signalling/2438	Rabbit	Western Blot- 1:1000
Anti-SREBP1	Abcam/ab3259	Mouse	Western Blot- 1:1000
Anti-L7α	Kind gift from Dr. Anne Roobol	Rabbit	Western Blot (used at supplied concentration)
Secondary Antibodies			
Anti-Mouse FITC conjugate	Sigma/F0257	Goat	Immunofluorescence 1:250
Anti-Rabbit FITC conjugate	Sigma/F9887	Goat	Immunofluorescence 1:250
Anti-Mouse HRP Peroxidase conjugate	Sigma/A4416	Goat	Western Blot- 1:2000
Anti-Rabbit HRP Peroxidase conjugate	Sigma/A6154	Goat	Western Blot- 1:2000

## 2 Experimental design, materials, and methods

### 2.1 Cloning and construction of vectors used for cell engineering and transient experiments

A summary of the details of vectors generated during this study is described in Table 1. The CHO specific *SCD1* gene sequence was amplified via polymerase chain reaction (PCR) using cDNA from Lonza's CHOK1SV™ cell line as the template and the primers 5′-TATGGTACCATGCCGGCC-3’ (forward) and 5′-ATACTCGAGCGGCTACTCTT-3’ (reverse). The *SREBF1* mouse gene sequence was isolated from an OriGene (Rockville, MD) derived vector bearing the cDNA clone for the mouse *SREBF1* gene (OriGene CAT no. MC205184, NCBI accession no. NM_011480) and amplified via PCR using the primers 5′-TATGCGGCCGCATGGACGAG-3’ (forward) and 5′-ATATCTAGACTGCTGGAAGTGACGGTGGTTC-3’ (reverse). *SCD1* or *SREBF1* genes were cloned into the pcDNA3.1V5-His/TOPO vector (Thermo Fisher Scientific) using *Kpn*I and *Xho*I or *Not*I and *Xba*I restriction enzymes respectively (as underlined in oligonucleotide sequences) in order to generate vectors with the capacity to overexpress the genes of interest which were in frame with the V5 tag in CHO cells. The *SREBF1* CHO cell gene sequence (NCBI accession no. NM_001244003.1) was synthesized by GeneART (ThermoFisher Scientific, USA).

Vectors were also constructed for the expression of model secretory recombinant proteins to assess the impact of cell line engineering on the secreted expression of these model molecules. In particular, we generated vectors from an in-house Lonza vector for a chimeric B72.3 (cB72.3) antibody as a model IgG4 molecule (considered to be easy to express by the authors) and a second Lonza vector was utilized which contained sequences for appropriate expression of a model Fc-fusion protein (FcFP). A vector for expression of a model IgG1 antibody (DTE-IgG1) considered by the authors to be difficult to express was also constructed. The construction of vectors for expression of an interleukin-2 fused bispecific antibody (IL2-F), containing the three genes required for expression of the IL2-F molecule, were built by having the individual genes synthesized by GeneART and then cloning these into a Lonza expression vector where each gene was under the control of a CMV promoter. Variations of the IL2-F vector were also generated such that they also contained sequences for appropriate expression of lipid metabolism modifying (LMM) genes *SCD1* (IL2-F-SCD1) or *SREBF1* (IL2-F-SREBF1), under the control of a CMV promoter, or a control (IL2-F-Control) lacking any LMM genes but inclusive of an empty expression cassette. All vectors contained a glutamine synthetase (GS) gene under the control of an SV40 promoter for use as a metabolic selection marker where necessary.

### 2.2 Cell culture and cell line construction

The process by which stably expressing SCD1 and SREBF1 cell pools and clones were established is described elsewhere [1] as is the process for generating stable recombinant protein secreting cell pool and lines. For the purpose of establishing the growth profiles of cell pools and lines under batch culture conditions, cultures were initially seeded with 0.2 × 10^6^ viable cells/ml in 20 ml of CD-CHO medium (ThermoFisher Scientific) and then cultured in a 5% CO_2_ balanced air environment in a shaking incubator at 140 rpm at 37 °C. Cell concentrations and culture viabilities were routinely determined using a ViCell (Beckman Coulter) instrument and 1 ml of culture sample. Culture viability was calculated as the number of viable cells as a proportion of total cells.

### 2.3 Transient expression of secretory recombinant biotherapeutic proteins

Transient transfections were performed using a GenePulser Xcell electroporator (Bio-Rad). Vector DNA (20 μg) diluted in 100 μl TE buffer and 700 μl of cells (1 × 10^7^ viable cells) were added to a cuvette. The DNA/cell mix was electroporated at 300 V and 900 μF in a cuvette with a 0.4 mm electrode gap. Medium (1 ml at 37 °C) was then added to the cuvette immediately after electroporation. The electroporated cells were then added to a flask containing 17.2 ml CD-CHO medium and a further 1 ml of medium was used to wash the cuvette and added to the culture such that a final volume of 20 ml was achieved in a 125 ml Erlenmeyer flask (Corning®). Cells were cultured in a 5% CO_2_ balanced air environment and batch transient cultures were incubated in a shaking incubator at 140 rpm at 37 °C.

### 2.4 Western blotting

Western blotting was undertaken as described elsewhere [2] and the details of the primary and secondary antibodies used in this study are outlined in Table 2. Quantitative densitometry was analyzed using *ImageJ* software.

### 2.5 Lipid extraction from cells and mass spectrometry analysis

The process for extracting lipids for analysis by mass spectrometry from cultured CHO cells and the process for the analysis of the mass spectrometry data is described in Ref. [1].

### 2.6 Immunofluorescence analysis

Cells were analyzed by immunofluorescence to determine the expression of exogenous ectopic SCD1 and SREBF1, and the localization of these proteins. The procedure for such analysis is described in Ref. [1] and the antibody details described in Table 2. Images were captured using a Zeiss LSM 880/Elyra/Axio Observer Z1 confocal microscope instrument.

### 2.7 Determination of secreted recombinant protein concentrations

Secreted recombinant protein concentrations in cell culture supernatants were determined using an Octet® instrument (ForteBio) with IgG calibrators and protein A biosensors.
